# dsRNA Molecules From the *Tobacco Mosaic Virus* p126 Gene Counteract TMV-Induced Proteome Changes at an Early Stage of Infection

**DOI:** 10.3389/fpls.2021.663707

**Published:** 2021-05-13

**Authors:** Naga Charan Konakalla, Mukesh Nitin, Athanasios Kaldis, Hema Masarapu, Sebastien Carpentier, Andreas Voloudakis

**Affiliations:** ^1^Laboratory of Plant Breeding and Biometry, Agricultural University of Athens, Athens, Greece; ^2^Department of Virology, Sri Venkateswara University, Tirupati, India; ^3^Department of Biosystems, KU Leuven, Leuven, Belgium; ^4^School of Life Sciences, Jawaharlal Nehru University, New Delhi, India; ^5^SYBIOMA: Facility for Systems Biology Based Mass Spectrometry, Leuven, Belgium

**Keywords:** double-stranded RNA, plant proteomics, RNA interference, silencing suppressor, *Tobacco mosaic virus*, tobacco

## Abstract

Exogenous application of double-stranded RNA (dsRNA) in the tobacco–*Tobacco mosaic virus* (TMV) pathosystem was shown previously to induce resistance against TMV providing an alternative approach to transgenesis. In the present study, we employed proteomics technology to elucidate the effect of TMV on tobacco as well as the effect of exogenous application of TMV p126 dsRNA molecules (dsRNAp126) at an early stage of the tobacco–TMV interaction. The proteome of tobacco leaf at 15 min post inoculation (mpi) in the presence or absence of dsRNAp126 molecules was studied. Thirty-six tobacco proteins were differentially accumulated in TMV-infected vs. healthy tobacco leaf tissue. The identified main differential TMV-responsive proteins were found to be involved in photosynthesis, energy metabolism, stress, and defense responses. Most of the virus-induced changes in the tobacco leaf proteome were not observed in the leaves treated with dsRNAp126 + TMV. The results indicated that the protein changes induced by TMV infection were counteracted by the exogenous application of dsRNAp126 molecules. Moreover, using small RNA sequencing, we showed that the exogenously applied dsRNAp126 was efficiently processed in tobacco as early as 15 min post application (mpa) to produce small interfering RNAs (siRNAs); the dicing pattern was not affected by the presence of TMV. The presence of dsRNAp126 reduced TMV p126 RNA abundance suggesting virus titer reduction *via* a sequence-specific mechanism, since a non-homologous dsRNA did not protect from TMV infection nor affect TMV accumulation.

## Introduction

RNA interference (RNAi) plays an important role in plant defense against subcellular pathogens including viruses (Padmanabhan et al., 2009; Wang M. B. et al., 2012). Double-stranded RNA (dsRNA) and stem-loop RNAs are the crucial players in RNAi initiation (Meister and Tuschl, 2004; Brodersen and Voinnet, 2006). The dsRNA-specific nucleases, known as DICER-like proteins (DCL), cleave the dsRNA into double-stranded small interfering RNAs (siRNAs) of 20–25 nucleotides in length; subsequently, one siRNA strand (guide strand) interacts with Argonaute (AGO) proteins resulting in the formation of the RNA-induced silencing complex (RISC), which facilitates target mRNA degradation (Dykxhoorn et al., 2003; Blevins et al., 2006; Deleris et al., 2006).

Viruses have developed a strategy to suppress RNAi, the most common way is to protect the viral genome against RNA silencing-mediated inactivation by encoding proteins that act as suppressors of RNA silencing (viral suppressors of RNA silencing, VSRs). VSRs inhibit the host silencing mechanism in several ways such as the inhibition of DICER protein activity and prevention of RISC assembly (Blevins et al., 2006; Brodersen and Voinnet, 2006; Wang M. B. et al., 2012). The severity of viral disease symptoms is usually correlated with the effectiveness of viral-encoded proteins to act as VSRs. Previous studies have shown that exogenous application of dsRNA targeted to VSRs conferred significant resistance against plant viruses (Tenllado and Díaz-Ruíz, 2001; Yin et al., 2009; Gan et al., 2010; Voloudakis et al., 2015; Konakalla et al., 2016; Kaldis et al., 2018). *Tobacco mosaic virus* (TMV) is well-known for its high replication rate and its capacity for rapid systemic movement in order to establish a successful infection. The p126 and p183 genes (components of viral replicase) get translated at a very early stage during TMV infection (Lewandowski and Dawson, 2000). Multiple domains of p126 protein especially methyltransferase, helicase, and non-conserved region II domains have independent host RNA silencing suppressor function (Wang L. Y. et al., 2012). Moreover, the p126 and p183 proteins alter the host metabolism in such a way to facilitate TMV replication within a few minutes upon viral entry into the host cell (Christensen et al., 2009; Niehl and Heinlein, 2011).

TMV p126 is an ideal target gene for dsRNA construction in order to apply the method of dsRNA vaccination for the induction of resistance against TMV in tobacco. Earlier, we reported the dsRNA-mediated virus gene silencing by the exogenous application of *in vitro*-produced dsRNA molecules (“RNA-based vaccination”) targeted to TMV p126 and TMV CP genes and demonstrated its role in minimizing TMV infection and disease induction (Konakalla et al., 2016).

The understanding of the complete mechanism underlying the events of virus–host plant interaction is crucial for the development of novel plant virus resistance strategies. TMV is a positive-sense, single-stranded RNA virus that belongs to the genus *Tobamovirus* of the *Virgaviridae* family. TMV has a very wide host range and its primary host is *Nicotiana tabacum*. It causes severe disease in tobacco with systemic mosaic symptoms that lead to a great reduction in quality and yield of tobacco leaves (Creager et al., 1999). Lee et al. (2006) employed two-dimensional gel electrophoresis (2DE) and revealed at 24 h post inoculation (hpi) that differentially accumulated proteins (DAPs) from TMV-inoculated *Capsicum annuum* leaves were involved in biotic stress, programmed cell death, metabolism, and mRNA binding. Two complementary proteomic methods DIGE and iTRAQ were employed by Caplan et al. (2009) to identify DAPs in *Nicotiana benthamiana* leaves in the absence (no defense response) and in the presence (defense response) of the *N* resistance gene. They revealed that the silencing of the endoplasmic reticulum (ER)-resident disulfide isomerases (NbERp57, NbP5) and the calreticulins (NbCRT2 and NbCRT3) leads to a partial loss of *N*-mediated resistance against TMV. An iTRAQ study of Wang et al. (2016) reported the effect of TMV on the proteome of two varieties of tobacco, namely NC89 (resistant to TMV) and its natural mutant Yuyan8 (tolerant to TMV). The authors concluded that differential proteins were respectively enriched in the photosynthesis and the pentose phosphate pathways.

Deep sequencing is a powerful means to study plant–virus interactions. In particular, the analysis of virus-derived small interfering RNAs (vsiRNAs) could reveal the hot (high number of vsiRNAs) and cold (low number of vsiRNAs) spots of the targeted virus genome indicating functional dicing by DCL proteins (Donaire et al., 2009). Qi et al. (2009) have obtained the small RNA profile of the TMV-Cg strain in *Arabidopsis thaliana* at 3 days post inoculation.

In the present study, *in vitro*-produced dsRNA molecules derived from the p126 gene of TMV were topically applied to the plants, as described previously (Tenllado and Díaz-Ruíz, 2001; Konakalla et al., 2016). The working hypothesis was that the synthesized dsRNAs (dsRNA vaccines) would induce the plant RNAi mechanism against TMV at very early stages of infection resulting in the degradation of the TMV genome, conferring plant protection. Here we used two high-throughput omics approaches, label- and gel-free proteomics and small RNA next-generation sequencing (small RNA NGS), to analyze the effect that dsRNAp126 exerts against TMV in tobacco plants at a very early stage of infection such as 15 min post infection (mpi) and to provide experimental evidence that the exogenously applied dsRNA is successfully diced as early as 15 min post application (mpa) by the endogenous RNAi machinery of the plant. Moreover, the results show that the exogenously applied dsRNAp126 is efficient in blocking TMV accumulation and counteracts the changes that TMV exerts on the tobacco proteome.

## Materials and Methods

### *N. tabacum* Growth Conditions

*N. tabacum* cv. Xanthi plants were grown under 25/22°C day/night temperature and 16/8 h light/dark cycles in the greenhouse facilities of KU Leuven, Belgium. Supplemental lighting of 14 W m^–2^ at the plant level was provided when solar radiation was below 250 W m^–2^ during the daytime. Six tobacco plants at the four-leaf stage (6 weeks old) were used for the inoculations in each treatment. The TMV strain used was TMV-vulgare DSMZ No. PV-0107 (Leibniz Institute DSMZ, Braunschweig, Germany) and was maintained on *N. tabacum* cv. Xanthi plants.

### *In vitro* Synthesis of dsRNA Molecules

The dsRNAp126 molecules [nucleotides 426–1,091 of TMV (Accession No. NC_001367.1)] were produced by a two-step PCR approach and *in vitro* transcription protocol reported by Voloudakis et al. (2015) and adopted by Konakalla et al. (2016; Supplementary Figure 1). In a similar manner, dsRNA molecules derived from the Helper Component-Proteinase (HC-Pro) gene of *Zucchini yellow mosaic virus* (ZYMV) were produced and used as the non-homologous control treatment. A Fisher Scientific Multiskan FC Reader (Thermo Fisher Scientific, United States) was used to estimate the concentration of total nucleic acids spectrophotometrically.

### Topical Application of dsRNAp126 + TMV on Tobacco Plants for Proteomic Analysis

The treatments used were as follows: (a) water (negative control), (b) TMV, and (c) dsRNAp126 + TMV. Each treatment consisted of six tobacco plants. The TMV treatment consisted of 16 μl of TMV-infected tobacco leaf sap extracted from 14-day post inoculation (dpi) TMV-infected tobacco leaves (inoculum) at 5 × 10^5^ dilution, according to Thornberry and Nagaich (1962) complemented with 4 μl of water. DsRNAp126 + TMV treatment was prepared by adding 4 μl of the *in vitro*-produced TMV p126 dsRNA (28.9 μg/μl) with 16 μl of TMV-infected tobacco leaf sap (5 × 10^5^ dilution). The negative control consisted of 20 μl of water.

Tobacco plants were inoculated by gently rubbing 20 μl of each treatment material into the fourth fully grown carborundum-dusted leaf. The treatment applications were performed during morning time (9:00–10:00 a.m.). The treated leaves were thoroughly washed three times using 0.05% Triton X-100 with 3 min interval between each wash, followed by a final wash with water. At 15 mpi, 300 mg of leaf tissue was collected from each of the inoculated plants (six individual samples per treatment) and immediately placed in liquid nitrogen for proteomic analysis. Symptom development was monitored until 20 dpi. Percentage of disease incidence was calculated based on the number of plants infected out of the total plants used for inoculation in each treatment (Supplementary Figure 1).

### Proteomics Analysis

For each treatment, six biological replicates were performed. The treated (local) leaf comprised the sample. Proteins were isolated by the phenol extraction protocol reported by Carpentier et al. (2005) and Buts et al. (2014). Samples were collected, ground in liquid nitrogen, and immediately placed in lysis buffer (100 mM Tris–HCl pH 8.3, 5 mM EDTA, 100 mM KCl, 1% DTT, 30% sucrose, and protease inhibitor cocktail Roche) before phenol extraction. The total protein concentration in each sample was estimated by 2D Quant kit (GE Healthcare, United Kingdom).

Twenty micrograms of total proteins from each sample were digested with trypsin (trypsin protease, MS grade, Thermo Fisher Scientific, United States) and desalted using Pierce C18 solid-phase extraction columns according to the manufacturer’s instructions (Thermo Fisher Scientific) and dried in the SpeedVac until dry and dissolved in 5% ACN (acetonitrile) and 0.1% formic acid. The digested and desalted samples were injected (0.5 μg/5 μl) and separated on an Ultimate 3000 UPLC system (Dionex, Thermo Fisher Scientific) equipped with an Acclaim PepMap100 precolumn (C18 particle size 3 μm, pore size 100 Å, diameter 0.075 mm, length 20 mm, Thermo Fisher Scientific) and a C18 PepMap RSLC (particle size 2 μm, pore size 100 Å, diameter 50 μm, length 150 mm, Thermo Fisher Scientific) using a linear gradient (0.300 μl/min). The composition of buffer A is pure water containing 0.1% formic acid. The composition of buffer B is pure water containing 0.08% formic acid and 80% ACN. The 0–4% fraction of buffer B increased from 0 to 4% in 3 min, from 4 to 10% in 12 min, from 10 to 35% in 20 min, from 35 to 65% in 5 min, and from 65 to 95% in 1 min and stayed at 95% for 10 min. The fraction of buffer B decreased from 95 to 5% in 1 min and stayed at 5% for 10 min. The Q Exactive Orbitrap mass spectrometer (Thermo Fisher Scientific) was operated in positive ion mode with a nanospray voltage of 2.1 kV and a source temperature of 250°C. Mass LTQ/FT-Hybrid ESI Pos. Mode Cal Mix (MS CAL5-1EASUPELCO, Sigma-Aldrich, United States) was used as an external calibrant. The instrument was operated in data-dependent acquisition mode with a survey MS scan at a resolution of 70,000 (FWHM at *m*/*z* 200) for the mass range of *m*/*z* 400–1,600 for precursor ions, followed by MS/MS scans of the top 10 most intense peaks with +2, +3, +4, and +5 charged ions above a threshold ion count of 16,0001e + 6 at 17,500 resolution using normalized collision energy of 25 eV with an isolation window of 3.0 *m*/*z*, apex trigger of 5–15 s, and a dynamic exclusion of 10 s (Thermo Fisher Scientific).

### Protein Identification and Quantification

The data of MS were acquired using Xcalibur 3.0.63 software (Thermo Fisher Scientific, United States). All the raw data obtained were converted into Mascot generic format (MGF) files by Proteome Discoverer version 1.4 (Thermo Fisher Scientific, United States). The mzxml-converted files were imported to Progenesis v4.1 software (Non-linear Dynamics, United Kingdom) for peptide normalization, alignment, and selection (ANOVA *p* ≤ 0.1). Protein statistics were applied to the summed, normalized, and selected peptide intensities. Spectra were identified using MASCOT v2.2.06 against the UniProt database taxonomy “*Nicotiana*” (6,793 accessions). The parameters employed to query were as follows: parent tolerance of 10 ppm, fragment tolerance of 0.02 Da, variable modification oxidation of M, fixed modification with carbamidomethyl C, and up to two missed cleavages for trypsin. The total protein false discovery rate was calculated in Scaffold 3.6.5 (Proteome Software, United States). Protein identifications were retained having at least one identified peptide with 95% confidence.

### Statistical Analysis of Proteomic Data

On the protein level, ANOVA and partial least square discriminant analysis (PLS-DA) were done using the NIPALS algorithm in Statistica 8.1 (StatSoft, United States).

### Gene Ontology Enrichment Analysis and Network Construction

Gene annotations were taken from UniProt. We built an in-house tool to perform Gene Ontology (GO) enrichment based on a user-defined subset of genes in the UniProt format https://labtrop.shinyapps.io/UniGO/. The tool is based on TopGO, an R package, and grasps the GO from the UniProt website and then performs a Fisher’s exact test to estimate the enrichment. To correct for multiple testing, the Holm–Bonferroni principle was applied. All ANOVA significant (*p* < 0.05) proteins were used as input for GO enrichment. The Excel file having the UniProt ID of each protein with corresponding GO terms (protein accession numbers in UniProt) was introduced to Cytoscape v3.4.0 for the construction of protein networks (Shannon et al., 2003).

### Topical Application of dsRNAp126 + TMV on Tobacco Plants for Small RNA NGS Analysis

The treatments used were as follows: (a) TMV, (b) dsRNAp126 + TMV, (c) dsRNAp126, and (d) water (negative control); there were six tobacco plants per treatment. TMV or dsRNAp126 + TMV application was performed as mentioned above. DsRNAp126 treatment consisted of only 4 μl of the *in vitro*-produced TMV dsRNAp126 with 16 μl of water. Tobacco plants were inoculated by gently rubbing 20 μl of each treatment material as mentioned above. Samples for RNA extraction were pooled from six plants in every treatment. Total RNA extraction from the local leaves of four treatments was carried out at 15 mpi using TRIzol (Life Technologies, United States). RNA extracts (3 μg) were sent for small RNA NGS analysis to Fasteris SA (Geneva, Switzerland). Upon small RNA library preparation, high-throughput sequencing was performed using the HiSeq 2500 Sequencing System (Illumina, San Diego, United States). Inserts with the size of 20–25 nucleotides were selected and mapped against a reference sequence for the TMV genome (Accession No. NC_001367.1) employing Bowtie v2.3 (Langmead et al., 2009) using default parameters. Further analysis and representation of the small RNA NGS data (hot and cold spots) was carried out using MISIS software (Seguin et al., 2014).

### Virus Titer Estimation *via* TMV p126 Expression Levels Made by RT-PCR

Reverse transcription (RT) quantitative PCR (RT-qPCR) was used for the estimation of TMV p126 gene abundance from H_2_ O-, TMV-, and dsRNAp126 + TMV-treated tobacco local leaves. RNA samples from two biological replicates were collected at two time points, e.g., 15 min and 24 h post treatment. Primers were designed outside the region of the p126 gene (426–1,091 of TMV genome) that was used for the production of dsRNAp126. For the RT, we employed the FIREScript Reverse Transcriptase (Solis BioDyne, Estonia). For qPCR, we used the 5 × HOT FIREPol^®^ EvaGreen^®^ qPCR Supermix (Solis BioDyne, Estonia) in a StepOnePlus Real-Time PCR System (Thermo Fisher Scientific, United States). The *C*_t_ values were normalized with a tobacco actin gene (Nt-ACT9, GenBank accession number: X69885.1) as an endogenous control. Quantification of TMV p126 was performed following the 2^–ΔΔ*Ct*^ method (Livak and Schmittgen, 2001). The sequences of primers for TMV p126 and Nt-ACT9 are shown in Supplementary Table 1.

## Results

### Proteomics Analysis of TMV-Inoculated Tobacco Plants in the Presence or Absence of dsRNAp126

**FIGURE 1:**
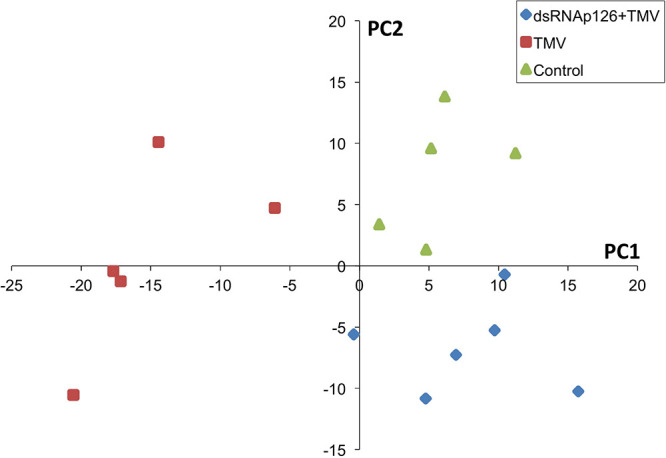
Partial least squares discriminant analysis (PLS-DA). Components 1 and 2 of the PLS-DA show a significant correlation to the treatments and explain, respectively, 24 and 8% of the variability. Component 2 separates the dsRNAp126 + TMV and control treatments from the TMV treatment. Each sample point is a biological replicate. All samples were collected 15 min post treatment.

For the investigation of early events occurring after TMV infection and in order to explore the putative effect of the application of dsRNA, we employed a proteomics approach. Protein samples from six biological replicates from TMV, dsRNAp126 + TMV, and control treatments (at 15 mpi) were used to perform the proteomics analysis. The mass spectrometric data showed that a total number of 661 proteins were confidently identified with a false discovery rate of 0.6% at the protein level and 0.04% at the spectrum level. To assess whether the proteome of the control, TMV, and dsRNAp126 + TMV samples was different, a PLS-DA statistical test was performed. Components 1 (PC1) and 2 (PC2) of the PLS-DA both show a significant correlation to the treatments and explain, respectively, 24 and 8% of the variability (Figure 1). Component 2 separates the TMV treatment from the dsRNAp126 + TMV and control treatments. This clearly indicates that TMV infection can already be detected as early as 15 mpi. Most importantly, the great majority of the TMV-induced changes in the tobacco leaf proteome were not observed in the leaves treated with dsRNAp126 + TMV (Table 1), suggesting that dsRNA treatment has a counteracting effect on the host proteome. Indeed, dsRNAp126 + TMV treatment showed 67% of resistance to TMV at 20 dpi (Supplementary Figure 1), in accordance with our previous work (Konakalla et al., 2016).

**TABLE 1:** List of differentially accumulated proteins (DAPs).

**No.**	**UniProt ID**	**Protein name**	**ANOVA *p*-value**	**Control^*a*^**	**TMV^*a*^**	**dsRNAp126 + TMV^*a*^**
**Proteins significantly more abundant in TMV treatment as compared with water and dsRNAp126 + TMV treatments**						
1	A0A077D0A9	Voltage-dependent anion channel	0.0003	b	a	b
2	E2F3S8	G-strand-specific single-stranded telomere-binding protein 1	0.0009	b	a	b
3	A0A075EZS9	Osmotin-like protein	0.0012	b	a	b
4	I2FJN7	Polyadenylate-binding protein (PABP)	0.0028	b	a	b
5	G3LV68	Chloroplastic NAD(P)H-quinone oxidoreductase subunit H	0.0029	b	a	b
6	A0A0F7R532	S-adenosylmethionine synthase	0.0073	b	a	b
7	Q6JE37	Thioredoxin-like protein CITRX2, chloroplastic	0.0081	b	a	b
8	Q40451	DNA-binding protein	0.0084	b	a	b
9	Q84UH4	Dehydroascorbate reductase	0.0125	b	a	b
10	J7G1D7	RNA-binding glycine-rich protein	0.0134	b	a	b
11	A0A075F933	Cysteine proteinase inhibitor	0.0171	b	a	b
12	B8R520	Small ubiquitin-related modifier (SUMO)	0.0175	b	a	b
13	Q5K4L4	Villin 2 (fragment)	0.0207	b	a	b
14	Q4LB98	Putative glutathione S-transferase (fragment)	0.0229	b	a	b
15	A0A0R4WFT2	Antimicrobial peptide	0.0234	b	a	b
16	A0A075F1V0	Malate dehydrogenase	0.0236	b	a	b
17	A2PYH3	Alpha chain of nascent polypeptide-associated complex	0.0281	b	a	b
18	H9CCI2	Acyl-carrier-protein S-malonyltransferase	0.0379	b	a	b
19	A0A075EYQ4	Ubiquitin-conjugating enzyme E2 35-like protein	0.0472	b	a	b
**Proteins significantly less abundant in TMV treatment as compared with water and dsRNAp126 + TMV treatments**						
20	A0A077DCK9	Carbonic anhydrase (carbonate dehydratase) (fragment)	0.0002	a	b	a
21	Q40565	Ribulose bisphosphate carboxylase/oxygenase activase 2, chloroplastic (RA 2) (RuBisCO activase 2)	0.0003	a	b	a
22	V9INR4	Ribulose bisphosphate carboxylase/oxygenase activase 2	0.0003	a	b	a
23	Q42961	Phosphoglycerate kinase, chloroplastic	0.0009	a	b	a
24	P06449	Cytochrome f	0.0011	a	b	a
25	A4D0J8	Carbonic anhydrase (carbonate dehydratase) (fragment)	0.0016	a	b	a
26	A0A075EYT9	Xyloglucan endotransglucosylase/hydrolase	0.0023	a	b	a
27	Q0PWS5	Chlorophyll a–b binding protein, chloroplastic	0.0032	a	b	a
28	P27141	Carbonic anhydrase, chloroplastic (carbonate dehydratase)	0.0053	a	b	a
29	A0A076KWG9	Chloroplast sedoheptulose-1,7-bisphosphatase	0.0061	a	b	a
30	A0A0E3JCP4	Developmentally regulated plasma membrane polypeptide	0.0071	a	b	a
31	Q9ZP50	FtsH-like protein Pftf	0.0079	a	b	a
32	A4D0J9	Carbonic anhydrase (EC 4.2.1.1) (carbonate dehydratase) (fragment)	0.0123	a	b	a
33	Q42962	Phosphoglycerate kinase, cytosolic	0.0175	a	b	a
34	P22302	Superoxide dismutase [Fe], chloroplastic (fragment)	0.0258	a	b	a
35	I0B7J4	Chloroplast PsbO_4_	0.0347	a	b	a
36	Q84N38	OBERON-like protein (Potyvirus VPg-interacting protein) (PVIPnb)	0.0414	a	b	a
**Proteins significantly more abundant in dsRNAp126 + TMV treatment as compared with water and TMV treatments**						
37	S6A7M4	Cysteine synthase (EC 2.5.1.47)	0.00004	b	b	a
38	Q3LAG5	Cysteine synthase (EC 2.5.1.47)	0.003	b	b	a
39	Q76MF3	Calmodulin	0.0212	b	b	a
40	E5LCN0	ACC oxidase 2 isoform A	0.023	b	b	a
41	Q76ME6	Calmodulin	0.026	b	b	a

**^*a*^Treatments with the same letter designation do not differ significantly at p < 0.05. a > b, n = 5–6.**

The differential abundance for the proteins was estimated based on ANOVA *p* < 0.05. Nineteen proteins were identified to be more abundant and 17 proteins were less abundant in the TMV treatment when compared with dsRNAp126 + TMV and water treatments (Table 1). The five most significant processes that were affected by the TMV infection were carbon utilization, generation of precursor metabolites and energy, photosynthesis, glycolytic process, and ATP generation from ADP (Supplementary Table 2).

**FIGURE 2:**
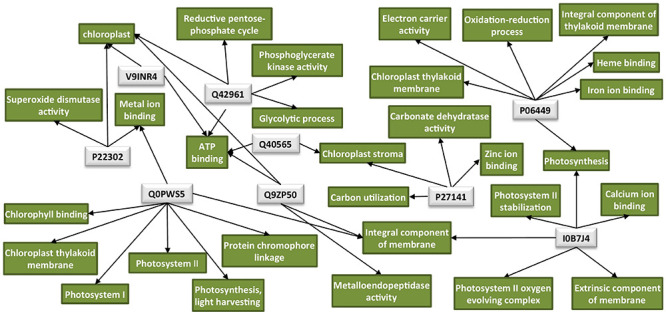
Protein network analysis of the chloroplastic proteins downregulated by TMV. Light-gray rectangles: UniProt protein IDs (Q40565: RuBisCO activase 2, V9INR4: RuBisCO activase 2, Q42961: phosphoglycerate kinase, P06449: cytochrome f, Q0PWS5: chlorophyll a–b binding protein, P27141: carbonic anhydrase, Q9ZP50: FtsH-like protein, P22302: superoxide dismutase [Fe], I0B7J4: PsbO4). Green rectangles: GO terms for each UniProt protein ID. GO IDs for individual GO terms could be found in Supplementary Table 3. Cytoscape was employed for the visualization of the network.

An overview of all significantly enriched GOs and all the annotated GOs for the DAPs is listed in Supplementary Tables 2, 3, respectively. Interestingly, the majority of the downregulated proteins in the TMV treatment were found to be associated with the chloroplast (Table 1). Network analysis revealed that the possible functions of these proteins are interconnected around the process of photosynthesis (Figure 2 and Supplementary Table 3).

Finally, the proteomics analysis resulted in a small number of proteins that are more abundant in the dsRNAp126 + TMV treatment when compared with the water and TMV treatments (Table 1). These were identified as 1-aminocyclopropane-1-carboxylate oxidase 2 isoform A (E5LCN0), cysteine synthase (Q3LAG5, S6A7M4), and isoforms of calmodulin (Q76ME6, Q76MF3).

### Small RNA Next-Generation Sequencing

**FIGURE 3:**
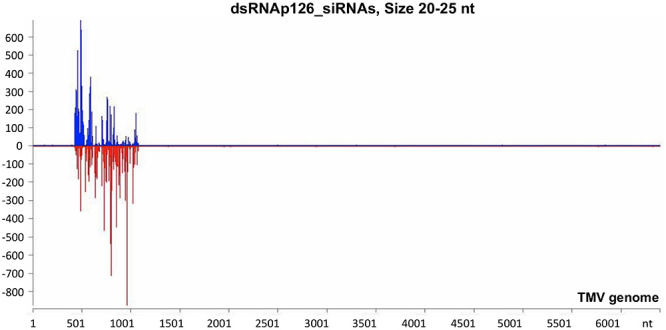
Analysis of siRNAs derived from the exogenously applied dsRNAp126 employing small RNA next-generation sequencing (NGS) as early as 15 min post application (mpa). Only dsRNAp126 was applied to tobacco plants and NGS analysis was performed at samples collected at 15 mpa. MISIS software visualization shows the distribution of siRNAs of 20–25 nt length along the sequence of a TMV reference genome (NC_001367.1). It could be observed that siRNAs are produced only in the region 426–1,091 of the TMV genome. On the *y*-axis are shown the total read counts of siRNAs. Sense strand reads are shown with blue color above the *x*-axis, and antisense strand reads are shown with red color below the *x*-axis.

The mechanistic basis of the protection induced by exogenous dsRNA application against TMV (Konakalla et al., 2016; this work) was studied by high-throughput sequencing (small RNA NGS). More precisely, we investigated whether dsRNAp126 molecules were diced in tobacco leaves treated only with dsRNAp126 in RNA samples collected at 15 mpa. We focused on the identification of siRNAs having 20–25 nt size because these siRNAs are considered to play an important biological role. The MISIS software was used to graphically represent the 20–25-nt siRNAs mapped along a reference TMV genome. As shown in Figure 3 and Supplementary Table 4, siRNAs derived from the targeted region of p126 (426–1,091 nt) were produced in very high quantities. The relative abundance of siRNA reads exhibits heterogeneity throughout the targeted region of p126, indicating that some regions can function as siRNA generating hot spots.

**FIGURE 4:**
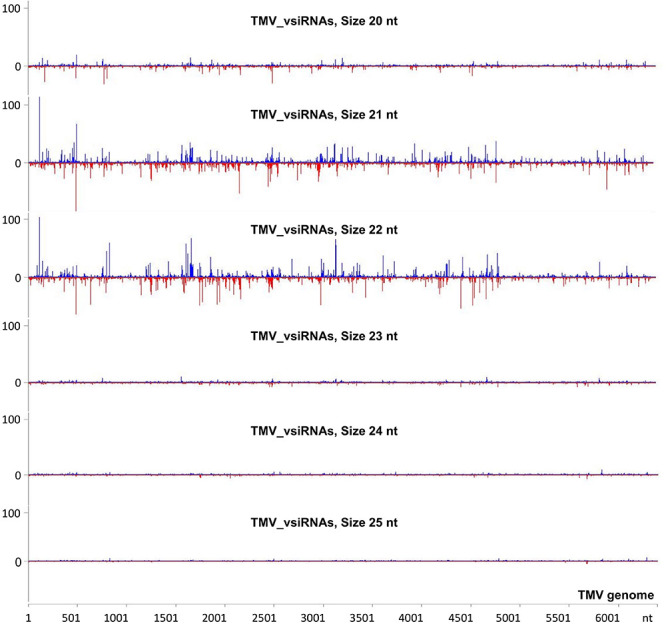
Analysis of TMV-derived siRNAs in infected tobacco at 15 min post inoculation (mpi) employing small RNA NGS. MISIS software visualization shows the distribution of virus-derived small interfering RNAs (vsiRNAs) of 20–25 nt length along the sequence of a TMV reference genome (NC_001367.1). On the *y*-axis are shown the total read counts of vsiRNAs. Sense strand reads are shown with blue color above the *x*-axis, and antisense strand reads are shown with red color below the *x*-axis.

To investigate the vsiRNA profile of the TMV genome at a very early stage of the infection, we used the small RNA NGS technique on RNA samples collected at 15 min post-TMV application. As shown in Figure 4 and Supplementary Table 5, TMV vsiRNAs of 21 and 22 nucleotides in length, spanning the entire TMV genome, are produced in much higher quantities when compared with vsiRNAs of 20, 23, 24, and 25 nt in length. The water treatment (negative control) sample had negligible siRNAs mapped to the TMV genome.

**FIGURE 5:**
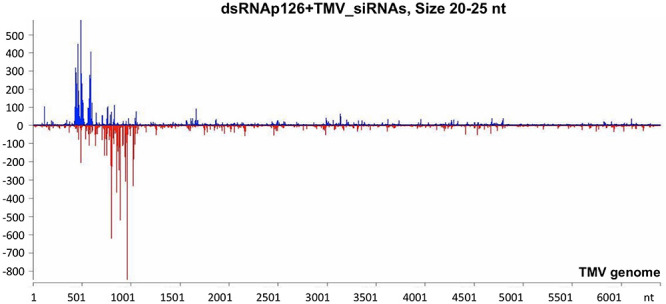
Analysis of siRNAs produced in dsRNAp126 + TMV treatment at 15 min post inoculation (mpi) employing small RNA NGS. MISIS software visualization shows the distribution of siRNAs having 20–25 nt length along the sequence of a TMV reference genome (NC_001367.1). On the *y*-axis are shown the total read counts of siRNAs. Sense strand reads are shown with blue color above the *x*-axis, and antisense strand reads are shown with red color below the *x*-axis. In the region 426–1,091 of the TMV genome, the majority of reads could be derived from the exogenously applied dsRNAp126. Outside this region, the reads represent TMV-derived siRNAs.

To investigate the siRNA profile of the TMV genome in case of the simultaneous presence of dsRNAp126 and TMV, we performed small RNA NGS on RNA samples collected from the dsRNAp126 + TMV treatment at 15 mpi. We observed that in the dsRNAp126 + TMV treatment, much higher quantities of siRNAs were produced from the targeted region of the p126 gene (dsRNA-derived and TMV-derived siRNAs) as compared with siRNAs outside this region (TMV-derived siRNAs) (Figure 5 and Supplementary Table 6). We noticed that the pattern of dsRNAp126-derived siRNAs is almost identical between the dsRNAp126 and dsRNAp126 + TMV treatments (compare Figure 3 with Figure 5; see also Supplementary Figure 2, where a representative region of the dsRNAp126 is presented).

It was also observed that in the 426–1,091 portion of the TMV genome, the profile of dsRNAp126-derived siRNAs is similar to that of TMV-derived siRNAs, e.g., the most highly abundant siRNAs are produced from the same hot spot regions in both the dsRNAp126 and TMV treatments (Supplementary Figure 3). This suggests that the dicing mechanism of the exogenously applied dsRNA possesses common components with the well-known sequence-specific dicing mechanism of the virus.

### Accumulation of TMV in TMV-Inoculated Tobacco Plants in the Presence or Absence of dsRNAp126

**FIGURE 6:**
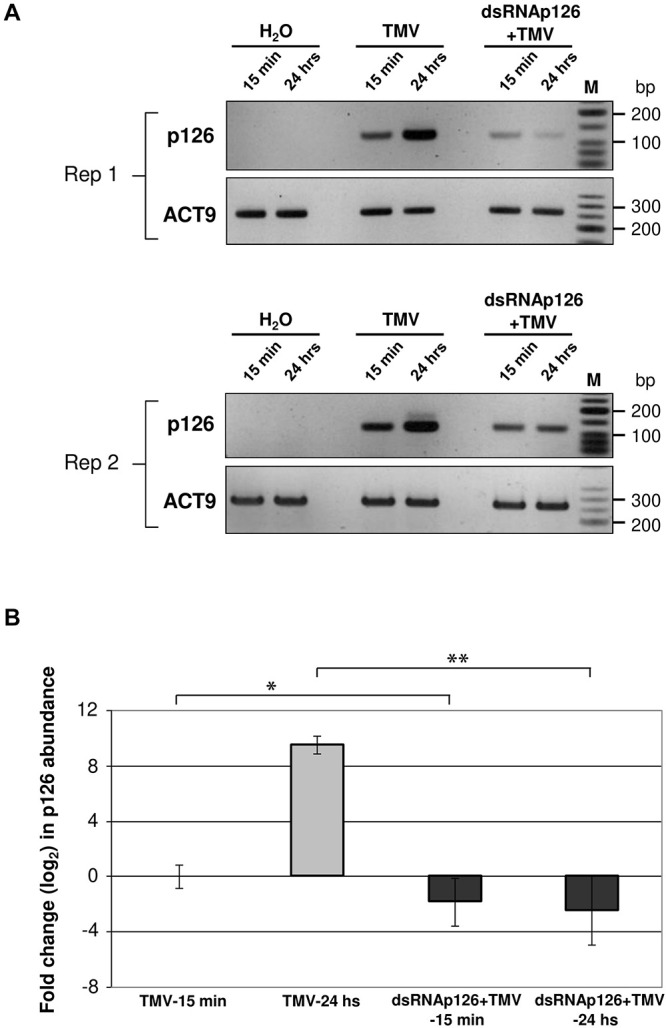
Analysis of TMV p126 expression levels in TMV and dsRNAp126 + TMV treatments by RT-PCR. **(A)** Gel images from two biological replicates (Rep 1 and Rep 2) showing p126 RNA abundance by semiquantitative RT-PCR. RNA samples were collected at two time points (15 min and 24 h post treatment). Primers for the detection of p126 were designed outside the region (namely 426–1,091 of the TMV genome) that was used for the production of dsRNAp126. Nt-ACT9 was employed as an endogenous reference gene. H_2_O indicates samples collected from the negative control treatment. M is a low molecular weight DNA marker (New England Biolabs, United States). **(B)** Relative quantification of p126 expression levels by RT quantitative PCR, employing Nt-ACT9 for normalization purposes. Depicted are the log_2_-transformed values of p126 abundance in TMV (light-gray columns) and dsRNAp126 + TMV (dark-gray columns) treatments. The value of p126 at 15 min in TMV treatment was set as 1 (log_2_ value was 0). Bars represent standard errors. Statistical analysis was performed per time point using Student’s *t*-test. Asterisks indicate significant differences between TMV and dsRNAp126 + TMV treatments at the respective time points (*p < 0.1; **p < 0.001).

DsRNAp126-derived siRNAs putatively could be loaded to AGO proteins of the host, functioning as efficient slicers of complementary TMV-derived RNAs, hampering as a result of the TMV-related processes inside the host cells or the TMV replication. To examine the effect of exogenous application of dsRNAp126 on TMV accumulation, we employed RT-qPCR to compare the p126 expression levels in TMV and dsRNAp126 + TMV treatments at two different time points, namely at 15 min and 24 h post treatment. At 15 min, p126 levels are lower in the dsRNAp126 + TMV treatment as compared with those in the TMV treatment. The difference in p126 levels between the two groups is more pronounced at 24 h (Figure 6A). For example, the log_2_ value of p126 abundance at 24 h in TMV treatment was 9.536, which corresponds to more than 700-fold induction relative to p126 amount at 15 min indicating a high TMV accumulation (Figure 6B). On the contrary, in the dsRNAp126 + TMV treatment, p126 abundance at 24 h remained low, as observed at 15 min (Figure 6B). This suggests that dsRNAp126 inhibits TMV accumulation, exhibiting as a result an antiviral effect (Supplementary Figure 1). To examine the specificity of this protective effect, we used dsRNA molecules produced for the HC-Pro gene of ZYMV as a non-homologous control. In accordance with our previous observations in other pathosystems (Kaldis et al., 2018), we found that non-homologous—to the targeted virus—dsRNA is not able to inhibit TMV accumulation and provides no resistance to TMV (Supplementary Figure 4). Taking these together, the above data suggested that the exogenous application of dsRNA is capable of inducing a sequence-specific defense mechanism.

## Discussion

Plants, as sessile organisms, need to respond to biotic or abiotic stresses by rapidly readjusting the abundance levels of specific proteins with a critical role to stresses. The orchestrated up- and downregulation of specific host factors in virus–plant interaction can result in a defense reaction against the virus. Alternatively, one could hypothesize that such alterations of the host factors will benefit the invading virus in a compatible interaction. The literature reports that protein changes are associated with a putative defensive role against TMV infection in tobacco, while other protein changes are associated with a successful TMV infection in tobacco-causing disease. Our proteomics analysis identified 661 proteins. Tobacco has an incomplete protein database to date (6,793 accessions) and a low annotated rate that could explain the low identification rate. The use of gel-free proteomics for protein identification requires a well-annotated database of the host species under study to be able to identify numerous proteins.

Despite the relatively low number of identified proteins, we found a significant correlation to the different treatments. Thirty-six (36) proteins were found to be differentially accumulated in the tobacco–TMV compatible interaction as early as 15 mpi. The protein changes that could contribute to resistance to TMV involve defense-related proteins such as S-adenosylmethionine synthase, cysteine proteinase inhibitor, glutathione S-transferase, malate dehydrogenase, Snakin-1, osmotin-like protein, and RNA-binding glycine-rich protein (upregulated upon TMV infection), as well as putative susceptibility factors such as phosphoglycerate kinases and OBERON-like protein (downregulated upon TMV infection) (Table 1).

S-adenosylmethionine synthase or SAMS (A0A0F7R532) catalyzes the formation of S-methylmethionine (SMM), which is necessary for the production of several osmoprotectant proteins (Espartero et al., 1994). Elevated levels of SMM could contribute to a response against plant stress, as it is a direct precursor of the osmoprotectant sulfoniopropionate family of proteins involved in both biotic and abiotic defense mechanisms. SMM also influences the biosynthesis of regulatory and defense compounds such as polyamines and ethylene (Tassoni et al., 2008). Cysteine proteinase inhibitor (A0A075F933) putatively plays a role in plant defense against viral infections (Shih et al., 1987; Prins et al., 2008). Gutierrez-Campos et al. (1999) expressed plant cysteine protease inhibitors in transgenic tobacco conferring resistance against potyviruses. Glutathione S-transferase or GST (Q4LB98) is involved in the neutralization of toxic compounds and reactive oxygen species formed during virus infection (Zechmann et al., 2005). In the case of *Bamboo mosaic virus*, plant GST binds to the viral RNA delivering glutathione to the viral replication complex (Chen et al., 2013). Glutathione is a key regulator of redox signaling and buffering and plays an important role in plant defense through the activation of defense-related genes (Foyer and Noctor, 2009). Malate dehydrogenase (A0A075F1V0) reversibly catalyzes the conversion of oxaloacetate to malate in both mitochondria and the cytoplasm, leading to the production of secondary metabolites (Tomita et al., 2005), such as alkaloids, flavonoids, and terpenoids, which are made in the reaction of mechanical damage or infection (Beggs and Wellman, 1994). The antimicrobial peptide Snakin-1 or SN1 (A0A0R4WFT2) has been associated with enhanced resistance against bacteria, fungi, and viruses (Almasia et al., 2008; Rong et al., 2013; He et al., 2017). The SN1 gene of soybean was found to enhance virus resistance in *Arabidopsis* and soybean probably by altering the expression of signal transduction and defense response genes (He et al., 2017). Osmotin-like protein (A0A075EZS9) belongs to the pathogen-related protein-5 (PR-5) family of proteins with a putative defensive role against several pathogens, besides its role as a osmoregulator. A higher abundance of osmotin-like proteins in TMV-infected leaf tissues has been reported (Broekaert et al., 1997). The glycine-rich RNA-binding protein or GRP (J7G1D7) participates in host–pathogen interactions, having a role in nucleic acid binding, hypersensitive response, and salicylic acid biosynthesis (Naqvi et al., 1998). Overexpression of the *A. thaliana* glycine-rich RNA-binding protein 7 (AtGRP7) conferred resistance against TMV (Lee et al., 2012). Furthermore, ADP ribosylation of the RNA-binding proteins attenuated host immunity by affecting RNA metabolism and the plant transcriptome related to defense (Fu et al., 2007).

Phosphoglycerate kinases or PGKs (Q42961, Q42962) were shown to promote the replication of several positive-strand RNA viruses (Cheng et al., 2013; Chen et al., 2017; Prasanth et al., 2017). This may be achieved either by their ATP-generating activity that facilitates the establishment of viral replication complexes (VRCs) (Prasanth et al., 2017) or by their viral RNA-binding capacity that helps the transport of viral RNA inside chloroplasts for replication (Cheng et al., 2013). Interestingly, a naturally occurring mutant of PGK in *A. thaliana* exhibits resistance to a potyvirus, suggesting that PGKs may function as host factors that increase susceptibility to virus infection (Ouibrahim et al., 2014). OBERON-like protein (Q84N38) was shown to promote systemic spreading of the potyvirus *Turnip mosaic virus* (TuMV) *via* interaction with the Vpg viral protein. Downregulation of OBERON-like protein decreases TuMV infection (Dunoyer et al., 2004).

All the above-described proteins, identified by proteomics analysis, are involved in the efforts of the host to defend itself against TMV at a very early infection stage, however, without success since plants are systemically infected by the virus. The protein changes correlated with a successful TMV infection involve the translation-associated poly-A binding protein (upregulated upon TMV infection), as well as chloroplast-associated factors such as carbonic anhydrases, chloroplast photosystem bO4 protein, and RuBisCO activase 2 (downregulated upon TMV infection) (Table 1 and Figure 2).

Poly-A-binding protein or PABP (I2FJN7) may act as a susceptibility host factor by promoting viral RNA translation (Iwakawa et al., 2012). TMV 3′ UTR contains several unique sequences designated as CAP-independent translation enhancer element (CITE) and A-rich sequences (ARS) which mediate viral RNA translation.

Carbonic anhydrase or CA enzymes (EC 4.2.1.1) (A0A077DCK9, A4D0J8, A4D0J9, P27141) have been identified as salicylic acid-binding proteins with an antioxidant role and generally constitute part of the defense mechanism in C3 plants upon attack by various pathogens, including viruses (Slaymaker et al., 2002; Restrepo et al., 2005). PsbO group protein D1 (I0B7J4) was shown to exhibit significantly lower abundance in TMV-sensitive tobacco cultivars upon TMV infection compared with the TMV-tolerant ones, indicating that high D1 levels are tightly correlated with antiviral resistance (Wang et al., 2016). In addition, silencing of PsbO in *N. benthamiana* resulted in increased TMV accumulation (Abbink et al., 2002). PsbO proteins are involved in the control of the photosystem II (PSII) affinity for manganese (Mn^2+^), thus participating in the stabilization of the oxygen-evolving complex (Popelkova et al., 2008). An intact and operational PSII complex is crucial for resistance to TMV infection (Wang et al., 2016). Interestingly, TMV p126 helicase protein interacts with a PsbO protein in a yeast two-hybrid system suggesting that p126 may interrupt PsbO’s normal localization and/or function (Abbink et al., 2002; Wang L. Y. et al., 2012). RuBisCO activases or RCAs (Q40565, V9INR4), being chaperone-like proteins, are required for optimizing photosynthesis and were found to colocalize with TMV replicase inside VRCs upon TMV infection. Downregulation of RCA substantially increases the infection of TMV (Bhat et al., 2013). Chloroplasts are energy generators, stress sensors, and defense signal producers and thus constitute major targets of invading viruses to establish successful infections (Li et al., 2016; Bhattacharyya and Chakraborty, 2018). Our findings showing the decrease in CA, PsbO, and RCA protein levels at 15 mpi could represent the beginning of a rapid alteration of the chloroplast caused by TMV (Table 1 and Figure 2). Typical TMV disease symptoms on tobacco leaves such as mosaic are correlated with abnormal chloroplast structure and distortion of the photosynthetic machinery (Lehto et al., 2003).

RNAi is considered a very effective antiviral mechanism (Padmanabhan et al., 2009; Wang M. B. et al., 2012). In addition, it was proposed recently that dsRNA could induce a pattern-triggered immune (PTI) signaling pathway in plants providing antiviral defense (Niehl et al., 2016 and references therein). No studies have been reported related to the production of vsiRNAs from the TMV genome as early as 15 mpi. Tobacco RNAi against TMV is operational at this time point in the tobacco–TMV interaction, since vsiRNAs from sense and antisense orientation of the TMV genome are produced (Figure 4 and Supplementary Table 5). However, even this defense reaction is not sufficient to confer resistance since all tobacco plants got infected. To boost the host RNAi machinery, we employed the topical application of dsRNAs, as inducers of the RNA-based vaccination (Voloudakis et al., 2018). In the present work, we used small RNA NGS to show that the exogenously applied dsRNAp126 on tobacco plants is efficiently processed as early as 15 mpa, producing siRNAs derived from the entire region of the dsRNA molecule used (Figure 3 and Supplementary Table 4), and this is to our knowledge reported for the first time. Since the siRNA profiles are similar in TMV and dsRNAp126 treatments (Supplementary Figure 3), we assume that DCLs play a key role in dicing the exogenously applied dsRNA. The observed heterogeneity in siRNA production (hot spots) (Figure 3) could be possibly attributed to the secondary structures of the dsRNA molecules that may restrict the accessibility of the DCL proteins to their targets. TMV *via* its p126 protein, which possesses RNA silencing suppressor activity, could interfere with the dicing capacity of the host DCL proteins, affecting as a result the exogenously applied dsRNA processing. However, this does not seem to happen in our experimental system. In particular, the simultaneous topical application of dsRNAp126 along with TMV produced the same pattern of siRNA molecules in the region 426–1,091 nt (region of dsRNA) (Figures 3, 5 and Supplementary Figure 2), suggesting that the presence of the virus does not alter the dicing pattern of the dsRNA applied. The siRNAs produced, at 15 mpi, in the region of dsRNAp126 (426–1,091 nt) is in great excess relative to the vsiRNAs derived from the rest of the viral genome (Figure 5 and Supplementary Table 6). This high abundance of dsRNA-derived siRNAs is a very important parameter for RNA-based vaccination since RNAi efficacy was previously shown to be dose dependent (Tenllado and Díaz-Ruíz, 2001). The efficacy of dsRNAp126 application against TMV infection can be illustrated by the finding that it significantly reduces the accumulation of TMV, as evidenced by RT-PCR analysis (Figure 6).

1-Aminocyclopropane-1-carboxylate oxidase 2 isoform A (ACO2) (E5LCN0), cysteine synthase (CyS) (Q3LAG5, S6A7M4), and isoforms of calmodulin (CaM) (Q76ME6, Q76MF3) compose a small group of proteins that were significantly more abundant in the dsRNAp126 + TMV treatment when compared with the water and TMV treatments. We hypothesize that these proteins are induced by dsRNA itself. No bibliographic reports exist involving ACO2 and CyS in plant virus infection. However, a calmodulin-like protein (rgd-CaM) suppresses the *Cucumber mosaic virus-2b* (CMV-2b), the viral silencing suppressor, by physical binding to CMV-2b’s dsRNA-binding domain (Nakahara et al., 2012). It would be important to further investigate the accumulation of these proteins to confirm their involvement in plant RNAi pathway and resistance to virus infection.

## Conclusion

The dsRNAp126 application has been shown repeatedly, in the experiments described here and in our previous study (Konakalla et al., 2016), to confer a significant level of protection against TMV. At the molecular level, dsRNAp126 is able to counteract the harmful effects of TMV on the chloroplast proteome (Table 1) as well as on PABP, a non-chloroplastic protein, very early in the tobacco–TMV interaction (Table 1). The observed counteracting effect by dsRNAp126 (based on the proteomics analysis) is suggested to be due to the lower TMV RNA (Figure 6) or TMV protein products (lower level of effectors) in the cell.

## Data Availability Statement

The datasets presented in this study can be found in online repositories. The names of the repository/repositories and accession number(s) can be found below: http://www.proteomexchange.org/, PXD022517; https://www.ncbi.nlm.nih.gov/genbank/, PRJNA665939.

## Author Contributions

NK performed the experiments, interpreted the results, and wrote the research manuscript. MN contributed to the bioinformatics analysis of small RNA sequencing data. AK performed the RT-qPCR analysis and assisted in writing the manuscript. HM contributed to the drafting of manuscript. AV and SC designed and supervised the present research work and corrected the manuscript. All authors contributed to the article and approved the submitted version.

## Conflict of Interest

The authors declare that the research was conducted in the absence of any commercial or financial relationships that could be construed as a potential conflict of interest.

## Supplementary Material

Supplementary Figure 1 DsRNAp126 production **(A)** and its protective effect against TMV infection in tobacco **(B)**.

Supplementary Figure 2 Highly similar pattern of dsRNAp126-derived siRNA production between dsRNAp126 and dsRNAp126 + TMV treatments.

Supplementary Figure 3 Similar pattern of dsRNAp126-derived and TMV-derived siRNA production in dsRNAp126 and TMV treatments, respectively.

Supplementary Figure 4 Non-protective effect of non-homologous dsRNAHC (deriving from HC-Pro of *Zucchini yellow mosaic virus*) against TMV in tobacco.

Supplementary Table 1 Primers for the amplification of TMV p126 and Nt-ACT9 in RT-qPCR.

Supplementary Table 2 An overview of all significantly enriched GOs.

Supplementary Table 3 An overview of all the annotated GOs for the differentially accumulated proteins (DAPs).

Supplementary Table 4 Next generation sequencing (NGS) data showing the production of siRNAs in the dsRNAp126 treatment at 15 min post application (mpa).

Supplementary Table 5 Next generation sequencing (NGS) data showing the production of siRNAs in the TMV treatment at 15 min post inoculation (mpi).

Supplementary Table 6 Next generation sequencing (NGS) data showing the production of siRNAs in the dsRNAp126 + TMV treatment at 15 min post inoculation (mpi).
