# Supplementary figures and images for: dsRNA Molecules From the Tobacco Mosaic Virus p126 Gene Counteract TMV-Induced Proteome Changes at an Early Stage of Infection
Source: Front Plant Sci. 2021 May 13;12:663707. doi: 10.3389/fpls.2021.663707 (PMC8155517; doi:10.3389/fpls.2021.663707)

**A**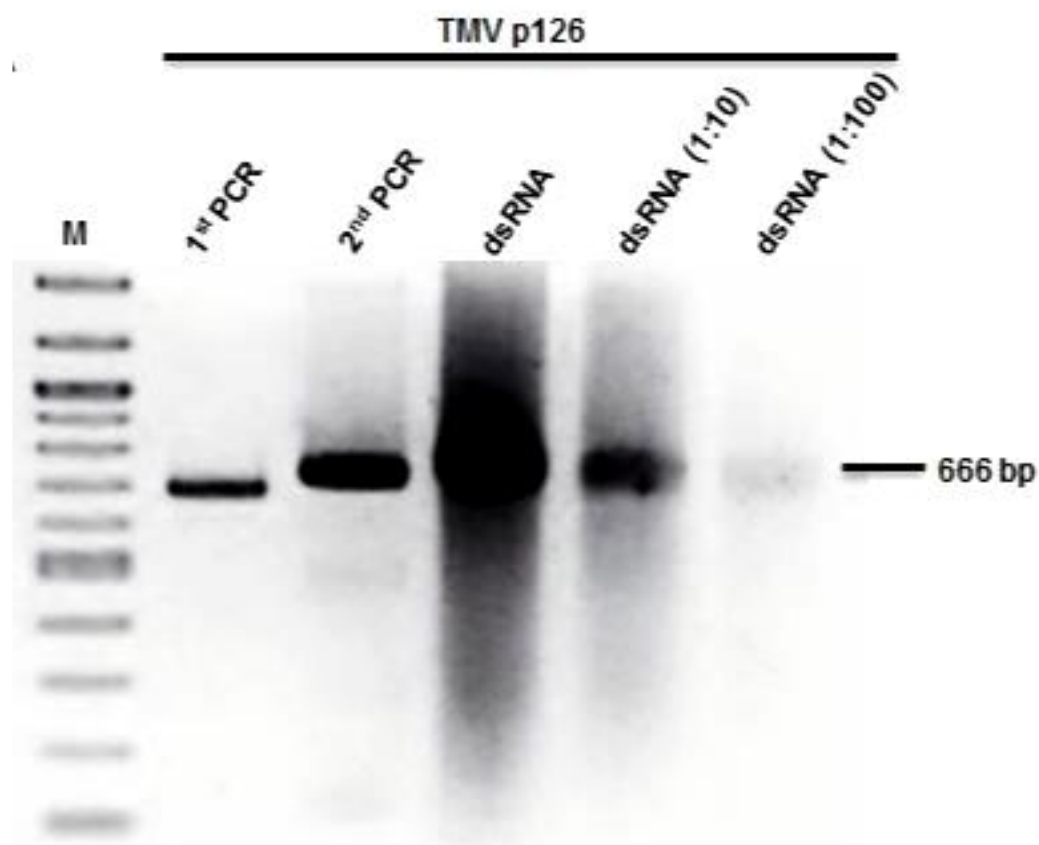**B**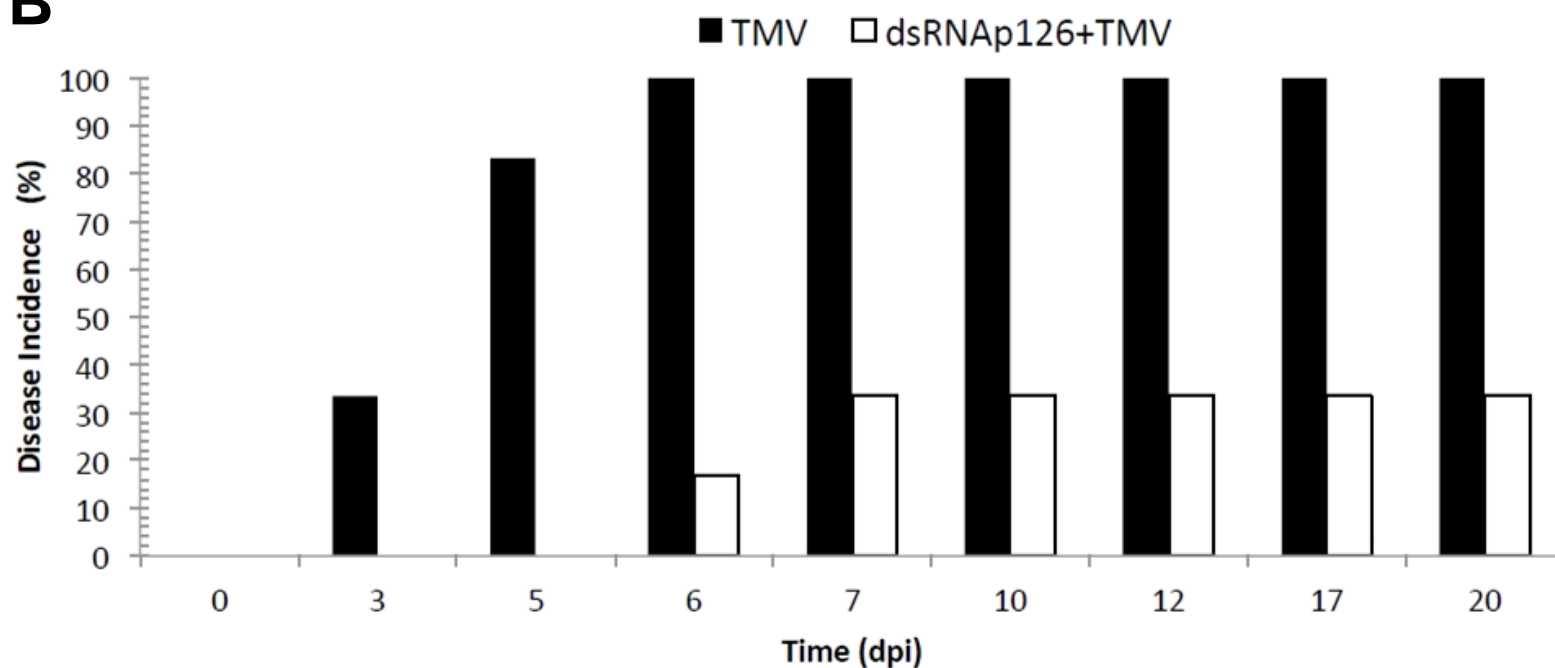

**FIGURE S1 |** DsRNAp126 production **(A)** and its protective effect against TMV infection in tobacco **(B)**.

Supplement: Supplementary Figure 1 — DsRNAp126 production (A) and its protective effect against TMV infection in tobacco (B). [file Image_1.pdf]
