# Supplementary material for: dsRNA Molecules From the Tobacco Mosaic Virus p126 Gene Counteract TMV-Induced Proteome Changes at an Early Stage of Infection
Source: Front Plant Sci. 2021 May 13;12:663707. doi: 10.3389/fpls.2021.663707 (PMC8155517; doi:10.3389/fpls.2021.663707)
Supplement: Supplementary Figure 2 — Highly similar pattern of dsRNAp126-derived siRNA production between dsRNAp126 and dsRNAp126 + TMV treatments. [file Image_2.pdf]

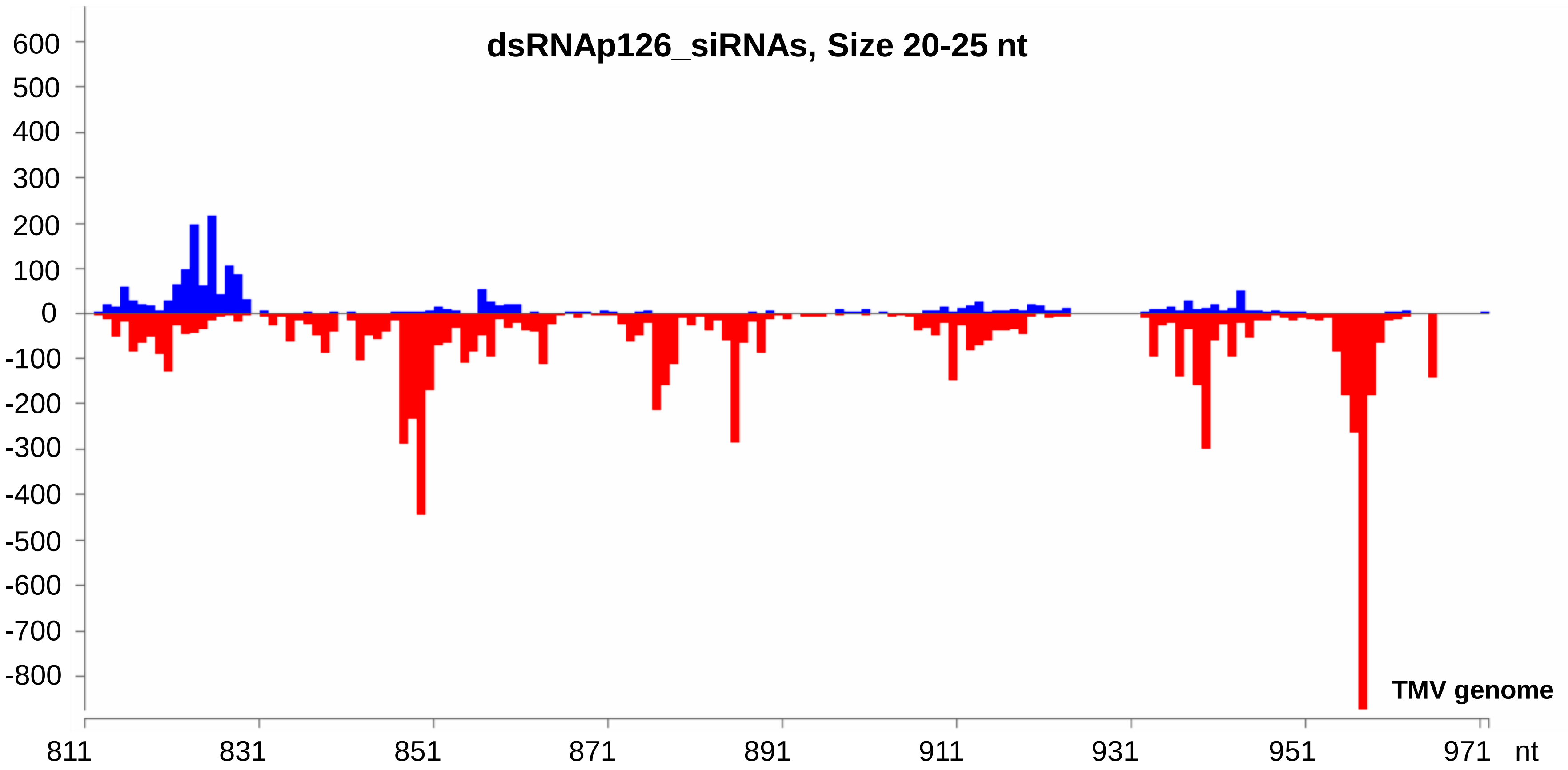

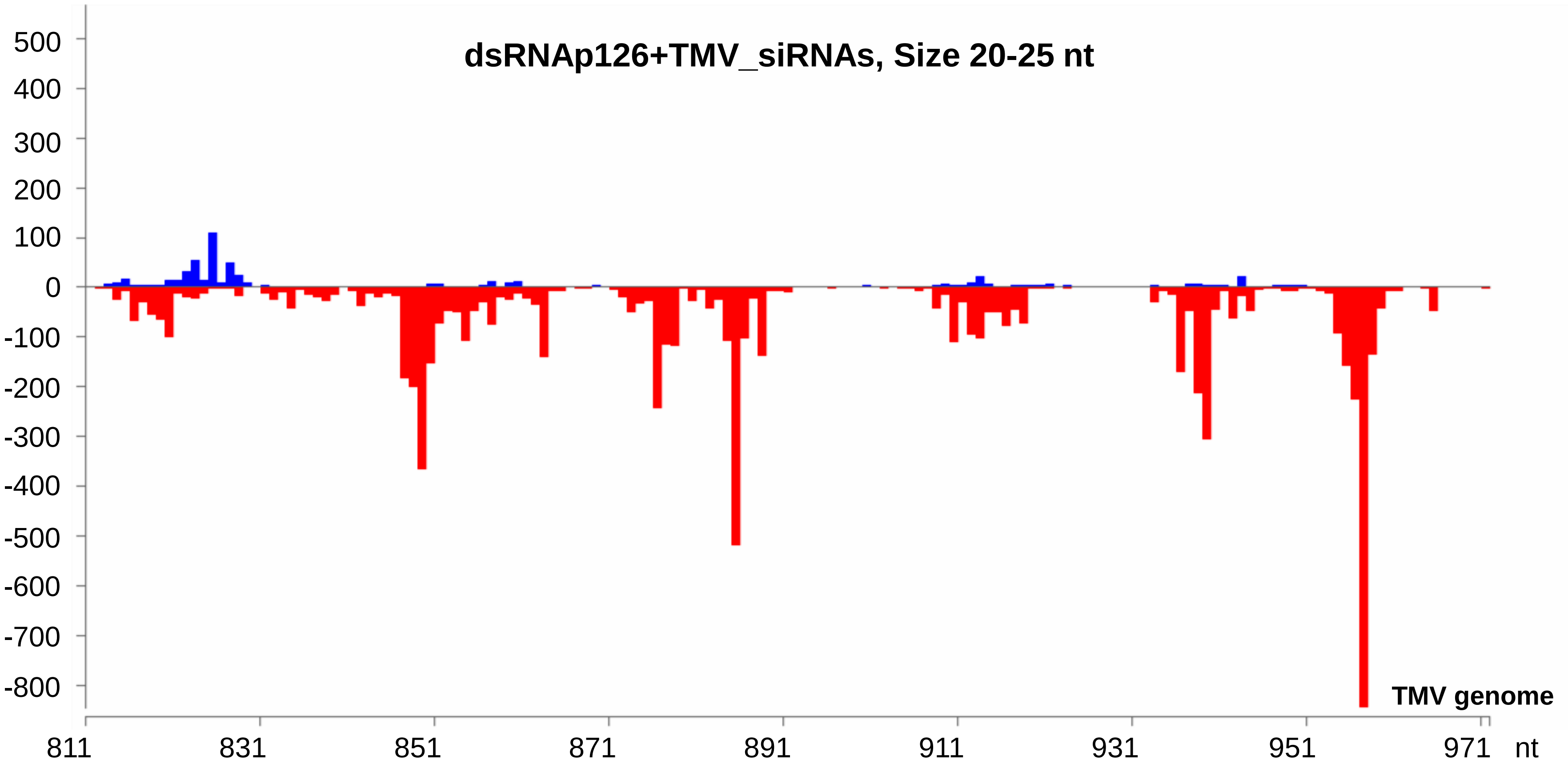

FIGURE S2 | Highly similar pattern of dsRNAp126-derived siRNA production between dsRNAp126 and dsRNAp126+TMV treatments. SiRNAs produced from a representative region (811-971 nt) of dsRNAp126 are shown. Upper panel: dsRNAp126 treatment; lower panel: dsRNAp126+TMV treatment.
