# Supplementary material for: dsRNA Molecules From the Tobacco Mosaic Virus p126 Gene Counteract TMV-Induced Proteome Changes at an Early Stage of Infection
Source: Front Plant Sci. 2021 May 13;12:663707. doi: 10.3389/fpls.2021.663707 (PMC8155517; doi:10.3389/fpls.2021.663707)
Supplement: Supplementary Figure 3 — Similar pattern of dsRNAp126-derived and TMV-derived siRNA production in dsRNAp126 and TMV treatments, respectively. [file Image_3.pdf]

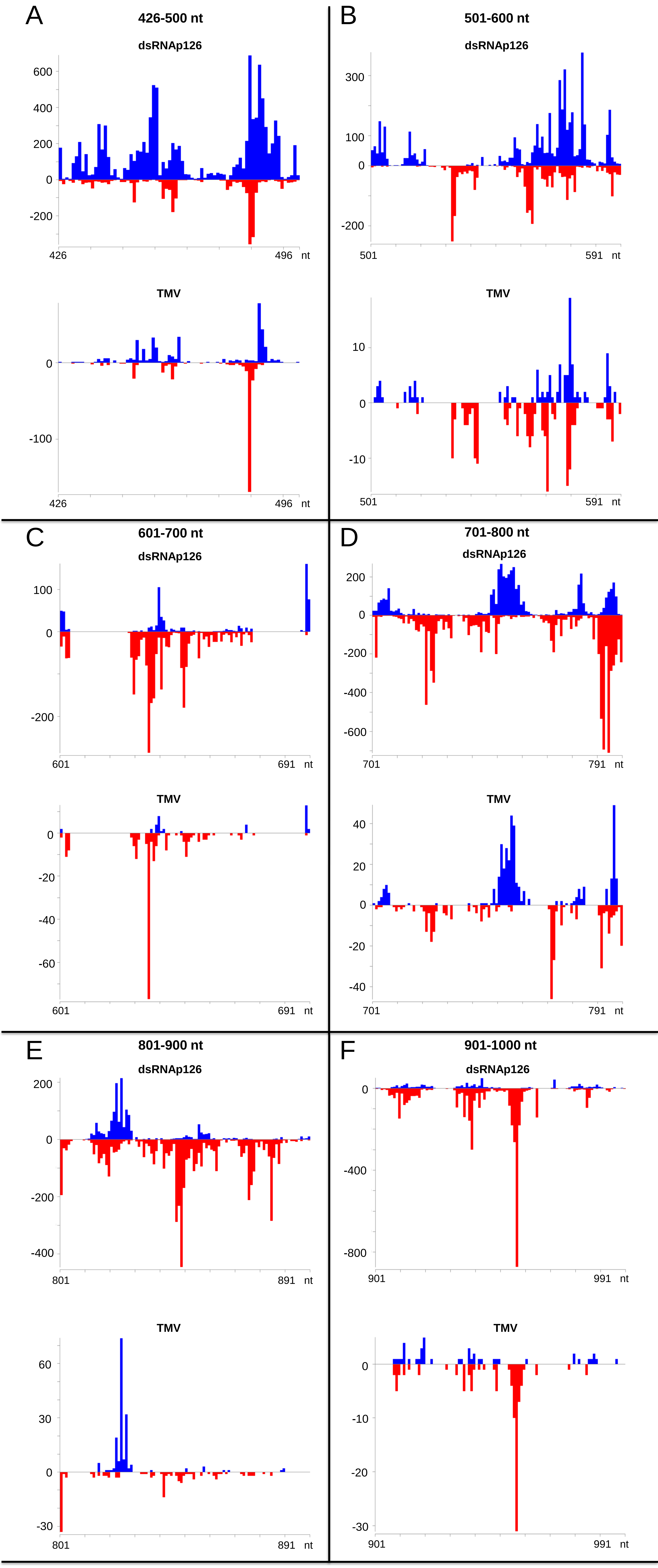

FIGURE S3 | Similar pattern of dsRNAp126-derived and TMV-derived siRNA production in dsRNAp126 and TMV treatments, respectively. SiRNAs produced from region 426-500 nt **(A)**, 501-600 nt **(B)**, 601-700 nt **(C)**, 701-800 nt **(D)**, 801-900 nt **(E)**, and 901-1000 nt **(F)**. The numbers derive according to the virus genome.
