# Supplementary material for: dsRNA Molecules From the Tobacco Mosaic Virus p126 Gene Counteract TMV-Induced Proteome Changes at an Early Stage of Infection
Source: Front Plant Sci. 2021 May 13;12:663707. doi: 10.3389/fpls.2021.663707 (PMC8155517; doi:10.3389/fpls.2021.663707)
Supplement: Supplementary Figure 4 — Non-protective effect of non-homologous dsRNAHC (deriving from HC-Pro of Zucchini yellow mosaic virus) against TMV in tobacco. [file Image_4.pdf]

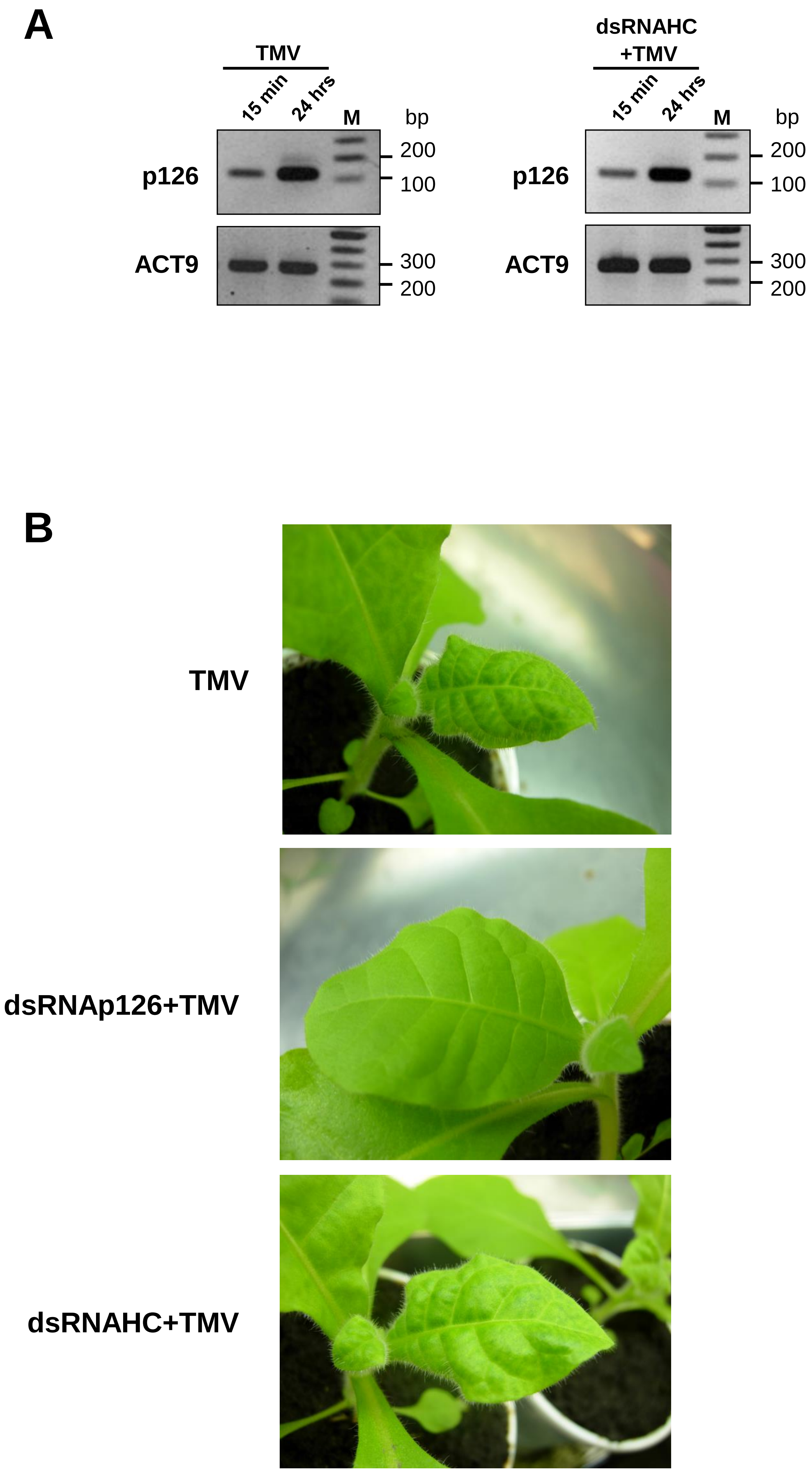

FIGURE S4 | Non-protective effect of non-homologous dsRNAHC (deriving from HC-Pro of *Zucchini yellow mosaic virus*) against TMV in tobacco. **(A)** Gel showing p126 RNA abundance by semi-quantitative RT-PCR. RNA samples from tobacco treated with TMV (left panels) and dsRNAHC+TMV (right panels) were collected at two time points (15 min and 24 hrs post treatment). Nt-ACT9 was employed as an endogenous reference gene. M is a low-molecular-weight DNA marker (New England Biolabs, USA). **(B)** Tobacco response to TMV (mosaic symptoms), dsRNAp126+TMV (no symptoms), and dsRNAHC+TMV (mosaic symptoms). Photographs were taken six days post treatment.
