# Supplementary material for: dsRNA Molecules From the Tobacco Mosaic Virus p126 Gene Counteract TMV-Induced Proteome Changes at an Early Stage of Infection
Source: Front Plant Sci. 2021 May 13;12:663707. doi: 10.3389/fpls.2021.663707 (PMC8155517; doi:10.3389/fpls.2021.663707)
Supplement: Supplementary Table 1 — Primers for the amplification of TMV p126 and Nt-ACT9 in RT-qPCR. [file Table_1.DOCX]

**TABLE S1 |** Primers for the amplification of TMV p126 and Nt-ACT9 in RT-qPCR.

| **Primer** | **Forward/ Reverse** | **Oligonucleotide sequence (5’- 3’)** | **Target** |
| --- | --- | --- | --- |
| qTMV_p126_1413F | F | CAATCCTTGTCCATGACGTTT | TMV p126 |
| qTMV_p126_1534R | R | GAAATCTCATCCCACACATGC | TMV p126 |
| Nt-ACT9-F | F | CTATTCTCCGCTTTGGACTTGGCA | Nt-ACT9 |
| Nt-ACT9-R | R | AGGACCTCAGGACAACGGAAACG | Nt-ACT9 |
